# Upward electrical discharges observed above Tropical Depression Dorian

**DOI:** 10.1038/ncomms6995

**Published:** 2015-01-21

**Authors:** Ningyu Liu, Nicholas Spiva, Joseph R. Dwyer, Hamid K. Rassoul, Dwayne Free, Steven A. Cummer

**Affiliations:** 1 Department of Physics and Space Sciences, Florida Institute of Technology, 150 West University Boulevard, Melbourne, Florida 32901, USA; 2 Space Coast Intelligent Solutions, Melbourne, Florida 32934, USA; 3 Department of Electrical and Computer Engineering, Duke University, Durham, North Carolina 27708, USA

## Abstract

Observation of upward electrical discharges from thunderstorms has been sporadically reported in the scientific literature. According to their terminal altitudes, they are classified as starters (20–30 km), jets (40–50 km) and gigantic jets (70–90 km). They not only have a significant impact on the occupied atmospheric volumes but also electrically couple different atmospheric regions. However, as they are rare and unpredictable, our knowledge of them has been built on observations that typically record only one type of such discharges. Here we report a close-distance observation of seven upward discharges including one starter, two jets and four gigantic jets above Tropical Depression Dorian. Our optical and electromagnetic data indicate that all events are of negative polarity, suggesting they are initiated in the same thundercloud charge region. The data also indicate that the lightning-like discharge channel can extend above thunderclouds by about 30 km, but the discharge does not emit low-frequency electromagnetic radiation as normal lightning.

## Abstract (web summary)

Upward electrical discharges from thunderstorms were discovered recently, and only a very limited set of observations exist because they are rare and unpredictable. Here, the authors present recordings of different types of the discharge above a storm, which contradict current theories of their origins.

## Graphical abstract

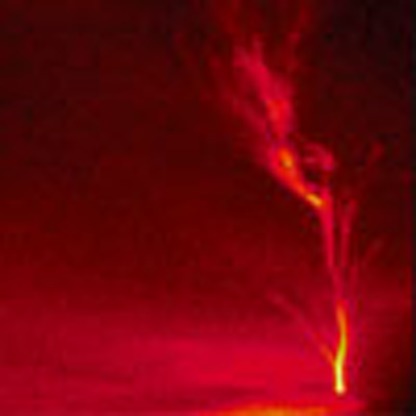

Upward electrical discharges from thunderstorms known as starters^1^,^2^,^3^,^4^,^5^, jets^4^,^5^,^6^,^7^,^8^ and gigantic jets^4^,^9^,^10^,^11^,^12^,^13^,^14^,^15^,^16^ belong to a larger group of electrical discharge phenomena in the middle and upper atmosphere caused by thunderstorm/lightning activities, which are termed transient luminous events^17^,^18^,^19^. Past observations indicated that starters and jets appear as a cone of blue light shooting upward from thunderstorms with a dimmer fan near their tops^1^,^3^,^4^,^5^,^6^, while gigantic jets display a tree-like structure and more complex dynamics^9^,^10^,^14^, and they bridge thunderstorms and the ionosphere, allowing a rapid transfer of a large amount of charge between the lower and upper atmosphere^12^,^16^. Most of the starters and jets reported to date occurred above land storms^1^,^3^,^4^,^5^,^6^,^7^,^8^, but gigantic jets predominately occur above tropical storms over oceans and coasts^20^,^21^.

The upward electrical discharges can be produced by thunderstorms through two principal mechanisms^2^,^22^. A standard, simple model of the charge structure of thunderstorms consists of two cloud charge layers of opposite polarities centred at different cloud altitudes and a screening charge layer around the cloud top that has the same polarity as the lower cloud charge. The upward electrical discharges can be developed from electrical breakdown, beginning either between the two cloud charge layers or between the upper cloud charge and the screening charge, where electric field is typically strongest. If a proper charge imbalance condition is created by electrical or meteorological processes, the initiated upward electrical discharge can penetrate through the charge layer it is directed to, and escape from the cloud top^2^,^22^. As the directions of the electric field are opposite at those two regions, the resulting upward electrical discharges have different polarities. This theory has been verified by observations reported later, indicating that the upward discharges beginning between the upper cloud charge and the screening charge tend to develop into starters or jets^3^, while those beginning between two cloud charge layers evolve into gigantic jets^12^,^16^.

The underlying electrical discharge process driving the development of starters, jets and gigantic jets is known as leaders^2^,^23^,^24^,^25^,^26^,^27^,^28^,^29^, the same as normal lightning. Leader discharges are responsible for electrically breaking down air to form a hot (>5,000 K), highly conductive channel, and their initiation and propagation mechanism is not well understood at present^30^. Metre-long leaders can be generated and studied in laboratory experiments. However, the kilometre-long leaders of natural electrical discharges possess significantly different characteristics, because the involved spatial and temporal scales are much larger and there are no well-defined counterparts of electrodes and discharge gaps as laboratory experiments. Observing various electromagnetic emissions from natural leaders using optical and radio instruments is the primary experimental means to study their discharge characteristics inside or outside thunderstorms^31^,^32^,^33^,^34^,^35^. From a theoretical perspective, the similarity laws of a particular electrical discharge or a particular stage of a discharge can be formulated and used if the same basic discharge processes dominate at different air pressures or densities^18^,^19^,^36^. Recently, theoretical studies have predicted that the leaders of the upward discharges propagate at a similar speed as lightning leaders, but require a significantly longer timescale to create a new section of the leader channel, which is found to be inversely proportional to the square of air density^26^,^27^,^28^,^29^.

Compared with normal lightning that frequently occurs during thunderstorms, starters, jets and gigantic jets are rare. Only one recent study reported an observation of all three types of the upward electrical discharges above a single storm, but the polarities of the events could not be unambiguously determined, because the storm was far away (~400 km) and electromagnetic measurements of the discharges were unavailable^4^. Here we present a close-distance (~80 km) observation of one starter, two jets and four gigantic jets above Tropical Depression Dorian. Our optical images and electromagnetic data indicate all of them are driven by negative leaders, suggesting that they originate between the two cloud charge regions. Our data also indicate that the leader channel above the cloud is charged similarly to a lightning leader channel, but it does not radiate low-frequency electromagnetic radiation as the lightning leader at lower altitudes. In addition, the upward leader can transfer a large amount of charge to the middle and upper atmosphere, even if it never reaches the ionosphere.

## Results

### The parent storm and lightning activities

The seven upward electrical discharge events occurred above Tropical Depression Dorian over the Atlantic Ocean between 3:45 Coordinated Universal Time (UTC) and 4:12 UTC on 3 August 2013. Tropical Depression Dorian formed from the remnants of the Tropical Storm Dorian, which started as a strong tropical wave off the west African coast on 22 July and evolved into Tropical Storm Dorian on 24 July when it was located about 300 km west of Cape Verde Islands en route to the southeast coast of United States^37^. Three days later, the storm weakened into a tropical wave, and on 2 August, when Dorian almost reached the coast of southeastern Florida, its remnants regenerated into Tropical Depression Dorian. In the early morning of 3 August, an hour before the first event, the ASCAT measurements from EUMETSAT Metrop-B Satellite indicated that the average wind speed of Dorian was 55 km h^−1^ with a maximum of 65–67 km h^−1^ in a localized area. Meanwhile, ground radar data from Melbourne, Florida, intermittently indicated that the wind speed was as high as 83 km h^−1^ at 1.5–1.7 km altitude on the south side of the circulation.

**Figure 1:**
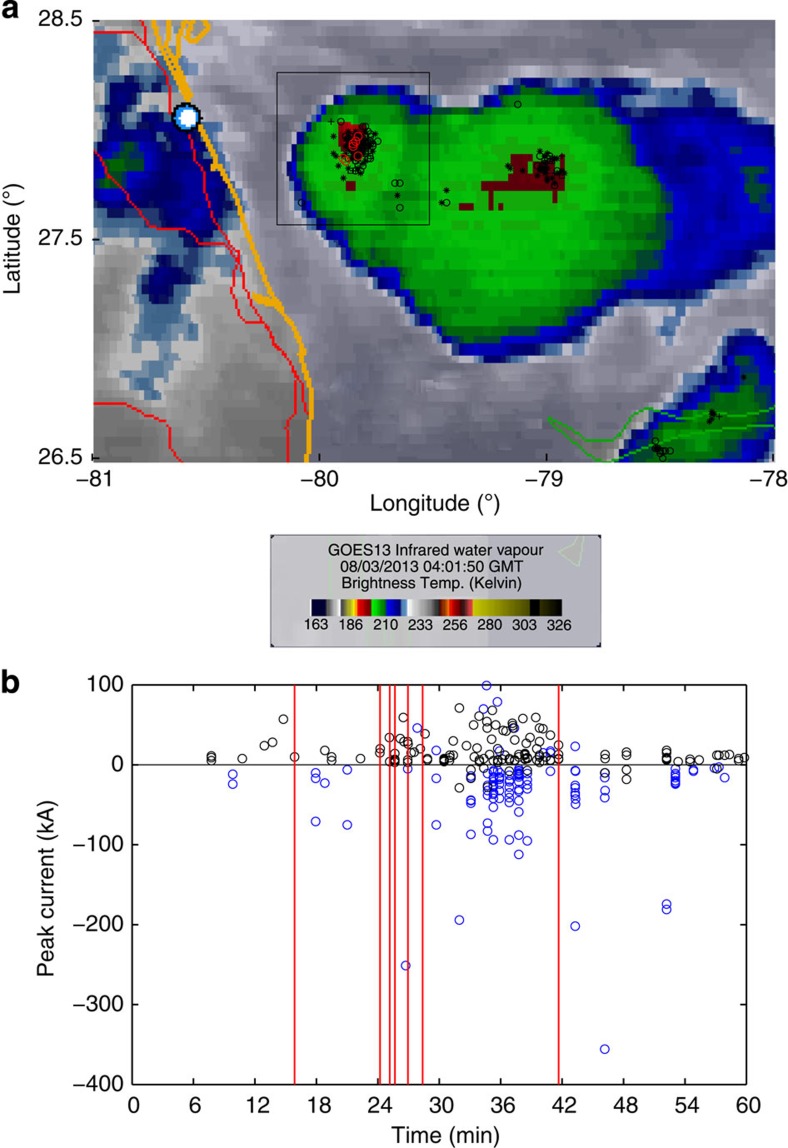
The parent storm and lightning activities. (**a**) The GOES infrared image of Tropical Depression Dorian that produced seven upward electrical discharges on 3 August 2013, on which the locations of the NLDN lightning events and the upward discharges are superimposed. Dark symbols represent the locations of the lightning events: ‘+’—positive CG lightning; ‘○’—negative CG; ‘*’—positive IC lightning; and ‘□’—negative IC. Red circles denote the locations of the upward discharges and the solid white dot represents the observation site. (**b**) A time scatter plot of the peak currents of the NLDN lightning events located within the black box in **a**. Black and blue circles represent ICs and CGs, respectively. The lightning activities are dominated by −CGs and +ICs, indicating that the parent cell was normally electrified. Vertical red lines show the occurrence times of the upward discharge events.

The GOES satellite infrared images from 2:45 UTC to 4:45 UTC show that several isolated small convection cells existed initially and they rapidly intensified, expanded and merged together. The entire storm also expanded rapidly, with its west edge reaching the east coast of Florida around 4:30 UTC. There were two active convection cores, when the events occurred. The one at the northwest corner of the storm, which was also closer to the observation site, was the parent cell producing the upward discharges. Figure 1a shows the GOES infrared image at 4:01 UTC, on which are plotted the locations of the lightning events recorded by the National Lightning Detection Network (NLDN^38^) from 3:30 UTC to 4:30 UTC. The cloud top temperature of the coldest area of that cell was 190–200 K and the NLDN lightning events clustered around that region. The open red circles denote the locations of the upward discharge events that overlap with the dense area of the NLDN lightning. Their distances to the observation site vary from 75 to 79 km.

Figure 1b shows a time scatter plot of the peak currents of the NLDN lightning events in the rectangular area (0.7° × 0.7°), covering the core of the parent cell, in Fig. 1a. There are a total of 266 NLDN events between 3:30 UTC and 4:30 UTC, including 12 positive cloud-to-ground (CG), 110 negative CG, 133 positive intracloud (IC) and 11 negative IC events. The polarity of a lightning flash is defined by the polarity of the charge that effectively moved downward. Most of the lightning events are −CGs or +ICs, indicating that the storm cell is normally electrified, that is, the main positive charge layer of the storm resides over its main negative charge layer^39^. The average NLDN lightning rate is ~4.5 events per min and the maximum average rate over a 5-min interval is 18.6 events per min, both of them falling in the normal range of thunderstorm cells^40^, (p. 25). As shown by the figure, the first event occurred in the early electrification stage of the cell, and 8 min later 5 events occurred in a 4-min interval. The last event occurred after the most electrically active stage of the cell, about 13 min apart from the previous event.

A more detailed analysis indicates that NLDN lightning events occurred in very close temporal and spatial proximity to each event, except the sixth event (a gigantic jet). However, it should be noted that NLDN lightning detection efficiency is not perfect^38^ and the video of the sixth event does show that lightning flashes occurred before and during this event. For the other six events, no NLDN events were found within a 20-s time period centred around each event, except a short time interval of 1–2 s containing the event. The fifth event (a gigantic jet) is the only one immediately before which CG activity was detected by NLDN. The sudden increase of the NLDN flash rate before each event is consistent with early studies of jets^7^.

### Video images of the events

**Figure 2:**
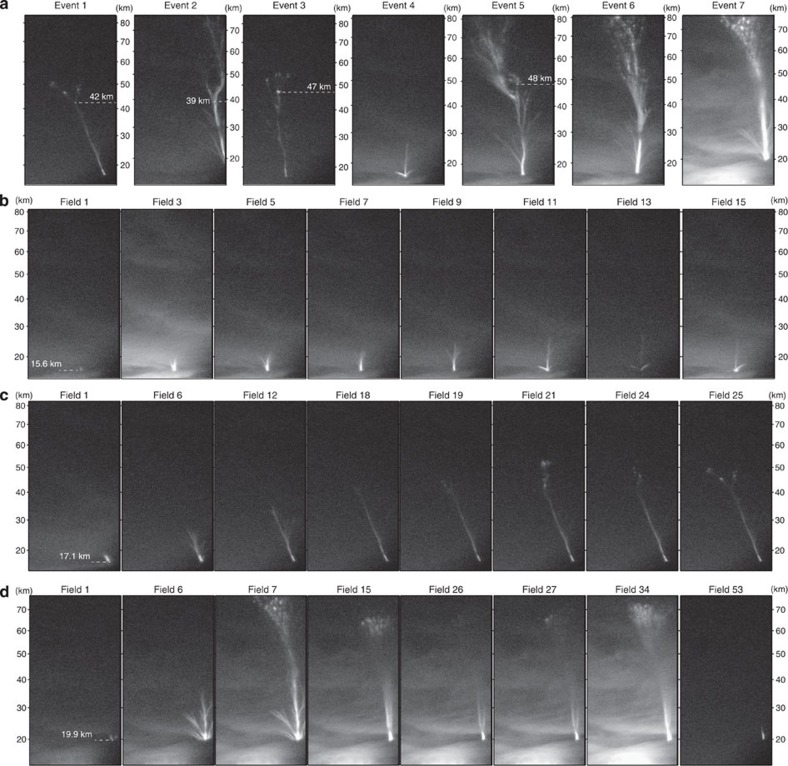
Low-light-level video fields of the seven upward discharges. (**a**) The seven events at their full extents. Events 1 and 3 are jets, event 4 is a starter and the rest of the events are gigantic jets. Selected video fields of (**b**) the starter (event 4), (**c**) a jet (event 1) and (**d**) a gigantic jet (event 7).

The upward discharge events were recorded by a low-light-level Watec camera and an all-sky camera installed on the campus of Florida Institute of Technology, and another all-sky camera about 10 km northwest. The recorded videos are available as Supplementary Movies 1–3. Figure 2 shows a few selected image fields (16.7 ms exposure time, Global Positioning System (GPS) time stamped) extracted from interlaced videos of 2–3 s for individual events recorded by the Watec camera. The seven events at their full extents are shown in Fig. 2a. The altitude labels to the right of each image vary from one event to another, because the distance of the jet to the camera is different. According to the videos, lightning flash(es) preceded every event and accompanied its development. Events 1 and 3 terminate at 51–55 km altitude, the tops of events 2, 5, 6 and 7 are outside the field of view of the camera, resulting in a >77–82 km terminal altitude, and event 4 terminates at ~26 km. On the basis of their significantly different terminal altitudes and temporal dynamics (as shown by the videos), we classify event 4 as a starter, events 1 and 3 as jets and the rest of the events as gigantic jets.

The video images show that all the events have a tree-like structure. For the starter and jets, they vanished in 50–60 ms after they reached their full extents, but the gigantic jets lasted much longer after their final jump to the ionosphere (that is, the sudden establishment of a discharge channel between the upward discharge and the ionosphere). For the two jets, after their main branches reached 42 and 47 km altitude, respectively, several branches were generated sequentially or simultaneously near their tops. The gigantic jets 2 and 5 initially propagated upward similarly as the jets. When they reached 39 and 48 km altitudes, respectively, multiple branches were produced at their tops similar to the jets, and then in the next video field one of those branches (event 2) or a branch below the top (event 5) made the final jump. Both events were followed by an intense lightning flash, which seems to fuel the short bases of the upward discharges to emit extremely bright light. The final jump was made at a lower altitude of 35 km from the tops of the upward discharges for events 6 and 7. After the final jump, the temporal dynamics were very similar for all gigantic jets, except that no visible lightning flashes followed events 6 and 7, and re-brightening of the discharge volume occurred for event 7. Compared with previously reported positive jets and starters^3^,^6^,^8^, the starter and jets have more branches and lack a diffuse fan top. This suggests that the upward leaders are of negative polarity, which is verified by the associated electromagnetic signatures (discussed below). The morphology and temporal dynamics of the gigantic jets are generally similar to the negative gigantic jets observed previously^9^,^10^,^14^. Figure 2b shows the detailed development of the starter, which lasted about 260 ms and had multiple branches connecting to a common, bright base (the multiple branches of the starter are more clearly shown by Supplementary Fig. 1 that presents a composite image of this event).

Figure 2c shows selected fields of event 1 that started around 3:45:51 UTC, following lightning flashes that began 150 ms earlier. The upward leader exited the cloud top at about 15.6 km altitude with a single main channel tilting from the vertical with an angle of 21°. For the next ~270 ms, the leader continued moving in that direction, while constantly spawning dimmer channels in a narrow cone of about 30°. Its vertical speed fluctuated between 4.5 × 10^4^ and 1.2 × 10^5^ m s^−1^ until its top reached 42 km altitude (fields 17 and 18), the uncertainty of which is ±4 km given that the leader channel might be tilted towards or away from the camera with an angle of 21° as well. The leader then appears unable to continue its steady propagation and dimmer channels originated from its top simultaneously and sequentially, as shown in the fields from 19 to 25. In field 19, a short, hardly visible vertical channel extended upward from the leader tip; it disappeared in the next field and then a small tree-like structure with a relatively larger vertical and horizontal extent suddenly appeared in field 21, resulting in a speed of 6.6 × 10^5^ m s^−1^ if it was formed by a discharge wave started from the leader tip. After field 25, the luminosity of the entire leader channel decreased rapidly and completely vanished in four fields. Given the different spatial structures and temporal dynamics of the discharges at the tip of the leader after it reached 42 km altitude, it is reasonable to speculate that the subsequent discharge activities near the leader tip show the streamer zone^27^,^28^,^29^ preceding the leader tip. If this is true, the vertical extent of the streamer zone is about 11 km for this particular leader tip at 42 km altitude. Following the same argument, the leader for event 3 reached an altitude as high as 47 km, as shown in Fig. 2a, which means that the leader channel extended a distance of more than 30 km above the thundercloud top.

Figure 2d shows the development of event 7, the most impulsive upward discharge event in our data set. The leader emerged from the cloud top with several distinct branches around 4:11:38 UTC. The centre branch had the highest top. Its vertical speed was initially 6.8 × 10^4^ m s^−1^ and then increased from 1.6 × 10^5^ to 2.1 × 10^5^ m s^−1^. It reached 34.8 km altitude in field 6, and then jumped to >77.1 km altitude in the next video field, resulting in a speed of >2.5 × 10^6^ m s^−1^. The speed and its variation with altitude are consistent with previously reported negative gigantic jets^9^,^14^,^27^,^29^. After the jump, relatively stationary bright beads and dimmer glows appeared at the top of the discharge. The luminosity of the top gradually decayed afterwards, while bead-like structures with short trails moved upward from about 50 km altitude along the pre-existing channels, as shown in field 15. The luminosity continued to decrease until field 26, when the top of the gigantic jet as well as the scattered light from cloud lightning activity started to re-brighten. The re-brightening reached its strongest stage in fields 34 and 35, which lasted 7 fields, and upward motion of the beads at the top as well as horizontal displacement of the entire discharge volume is visible. After the main body of the gigantic jet vanished, a short bright column base above the cloud, as shown in field 53, persisted for a while, and the entire duration of the discharge was as long as 1.2 s. To the best of our knowledge, this is the longest duration of the upward cloud discharges that have ever been reported.

### Magnetic field measurements and source waveforms

**Figure 3:**
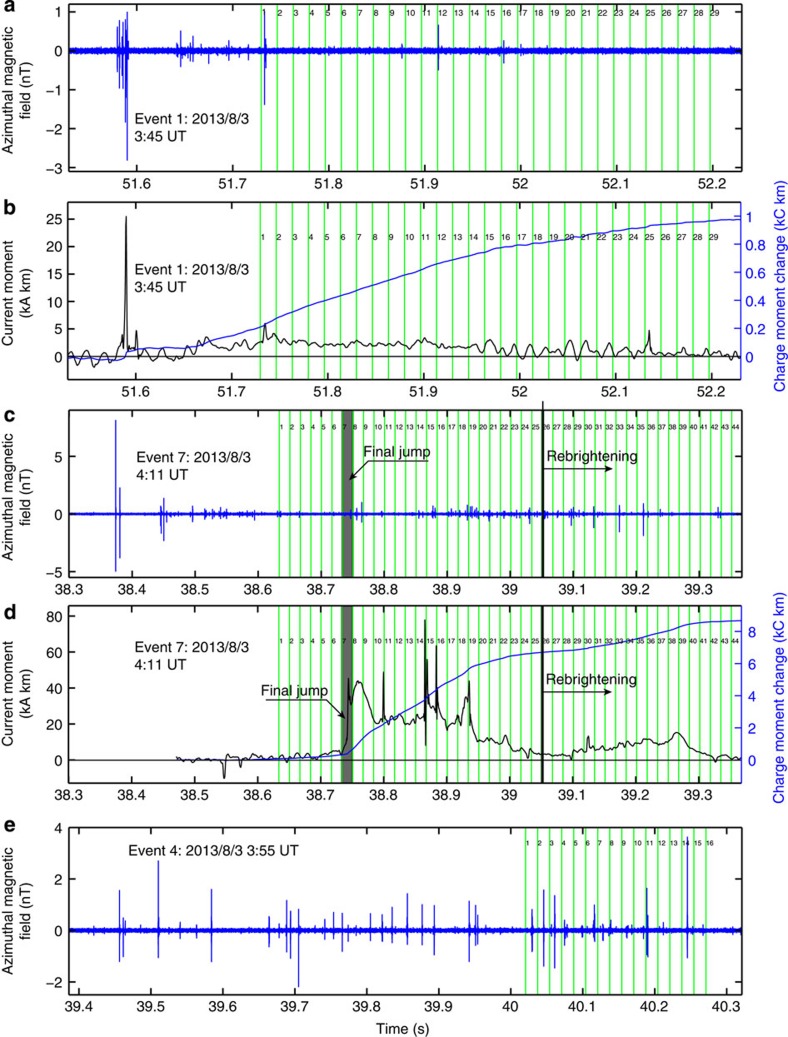
Magnetic-field measurements and source waveforms. (**a**,**b**)The LF magnetic-field waveform and the source waveforms (current moment and charge moment change), respectively, for the jet; (**c**,**d**) the same waveforms for the gigantic jet; and (**e**) the LF waveform for the starter. The current moments and charge moment changes are derived from the ULF measurements. Each vertical strip bounded by two green lines corresponds to an video field, with its field number given between the two boundary lines. The grey strips in **c** and **d** correspond to the video field showing the final jump.

The electromagnetic radiation from the upward discharges and accompanied cloud discharges was measured by a low frequency (LF) magnetic field detector at Florida Institute of Technology and by a ULF (ultralow frequency) magnetic field sensor at Duke University^12^,^16^. Figure 3 shows the magnetic field measurements of a jet (event 1 in Fig. 2a,c), a gigantic jet (event 7 in Fig. 2a,d) and a starter (event 4 in Fig. 2a,b). For all seven events, strong LF pulses, as shown in Fig. 3a,c,e, started to appear about 0.2–1 s before the upward discharges emerged above the cloud. Such LF pulses are known to be produced by in-cloud discharges^12^,^16^. For some events, the LF pulses started with a large narrow bipolar pulse with a width of 10–20 μs, similar to the first pulse in Fig. 3c, which has recently been shown to be associated with the initiation of the in-cloud discharges that led to two gigantic jets^16^. Additional electromagnetic radiation data collected by Kennedy Space Center indicate that the onset altitudes of the in-cloud discharges resulting in all the upward discharge events varied between 12–14 km altitudes.

Figure 3a,c show that there are no persistent LF activities during the propagation of the upward leader above the cloud. This indicates that the negative leader above the cloud did not radiate strong-enough LF pulses to be detected by the LF sensor ~80 km away, and that the cloud discharges were not as active as the early initiating stages of the upward discharges. However, the discharge activity, as indicated by the LF pulses, associated with the starter was continual before and throughout the event, suggesting the starter occurred during the active stage of the cloud flash. Figure 3a also shows that there are no strong LF signatures associated with the sequence of the discharge events occurring at the negative leader tip from the video field 18 to 25. It is known that the propagation of negative leaders consists of discrete steps and LF pulses are produced by the stepping process of the negative leaders of IC and CG lightning. The absence of LF activities suggests that the stepping of negative leaders above thunderclouds occurs on a longer timescale than the sensitive range of the LF sensor, possibly resulting from a larger spatial scale of the discharge at higher altitudes as suggested by the scaling laws of electrical discharges in air^28^,^29^,^41^. Figure 3c also shows that several distinct LF pulses appear right after the final jump of the upward discharge, which are probably produced by the rejuvenation of the cloud discharges due to the established electrical connection between the thundercloud and the ionosphere^16^.

Previous studies have demonstrated that gigantic jets can transfer a significant amount of charge from thunderclouds to the ionosphere^10^,^12^,^13^,^16^. All four gigantic jets observed carried strong-enough currents so that the associated current moment waveforms can be unambiguously determined from the ULF data. The resulting total charge moment change varies from 3.1 to 8.7 kC km and the total deposited charge in the middle and upper atmosphere varies from 48 to 134 C if a channel length of 65 km is assumed. The wide range of the total charge moment change agrees with an ensemble built from previous, separate observations^10^,^12^,^13^,^16^. The charge moment change can also be unambiguously extracted for a jet (event 1) to be 0.98 kC km, corresponding to 56 C charge transfer if the channel is assumed to be uniformly charged and have a length of 35 km, but not for the other jet (event 3) and the starter (event 4). Overall, the amount of charge moved upward from the thunderstorm by all the discharges in about 30 min is at least 383 C.

Figure 3b,d show the waveforms of the current moments and charge moment changes of event 1 and event 7. Figure 3b shows that the current carried by the upward leader of the jet begins to exceed the detectable level more than 100 ms before the first video field of the jet. The current moment stays at an approximately constant value of 1.5–2.5 kA km from 51.75 to 51.95 s during the upward propagation of the leader, which means that the current decreases as the leader propagates upward. Given the measured total charge moment change is 0.98 kC km for this event and there is only a single leader channel, the linear charge density of the channel can be estimated to be about 1.5 mC m^−1^, assuming that the charge is uniformly distributed along the channel and the channel length is 35 km, which is consistent with the linear charge density of a lightning leader^40^, (p. 123–126).

For the gigantic jet’s waveforms shown in Fig. 3d, the initial current moment up to field 5 is relatively small and the resulting charge moment change increases slowly to about 0.4 kC km, comparable to the final value of event 1. During the video field showing the final jump, the current moment of the gigantic jet rapidly increases to about 40 kA km, consistent with previous work^11^,^12^,^16^. The current moment maintains at such a high level for 30 ms, and then decreases to 20 kA km and stays there for the next 160 ms. About 65% (85 C) of the total amount of charge transferred between the thunderstorm and the ionosphere by this event occurs during this ~200 ms period. The re-brightening is accompanied by an increase in the current flowing in the discharge channel, resulting in a charge moment change of 1.8 kC km (21% of the total charge moment change of the event). The other gigantic jets (without re-brightening) have similar current moment and charge moment waveforms up to the moment of re-brightening, with the charge moment change before the final jump varying in the range of 0.3–1 kC km.

The negative polarity of the jet and the gigantic jet is unambiguously shown by the current moments and charge moment changes derived from the ULF magnetic field measurements. Although a reliable current moment waveform cannot be derived from the ULF data for the starter event, the conclusion of its negative polarity can be drawn based on the following reasons. As discussed above, its morphology is different from previously reported positive starters. Supplementary Fig. 2 shows that there are no signatures of downward negative leaders that would be expected to accompany a starter of positive polarity. The interpretation of negative polarity is consistent with fractal modelling results, as discussed in Supplementary Note. Finally, previous studies on the downward ‘attempted leader’ between thunderstorms and ground^42^ indicated it is possible for a negative leader that is rooted in positive leaders in the main negative cloud charge region not to propagate far after escaping from thunderstorms.

## Discussion

Event 1 in our data set is quite unique, because there is only a single leader discharge channel. The agreement between its linear charge density with typical values of lightning leaders provides an important new piece of evidence for the theory of jets and gigantic jets being escaped leaders from thunderstorms^2^,^22^,^24^,^25^. The two escaping mechanisms for the upward leaders^2^,^22^ have been verified recently^3^,^12^,^16^. Our observation further indicates that starters, jets and gigantic jets can be developed from leaders initiated from the same region in the thundercloud charge structure, that is, between the two main thundercloud charge reservoirs. Below, we offer an explanation why an upward leader develops into a starter/jet or a gigantic jet through examining the characteristics of the leaders, resulting in events 1 and 7. From the leader theory, the electric potential difference between the leader tip and the ionosphere can be determined if its altitude and streamer zone size are known^24^,^25^,^28^,^29^, assuming that the electric field in the streamer zone is the critical field for streamer propagation. If this field is assumed to be the critical field for negative streamer propagation, which is about two to three times larger than that field for positive streamers, the current derived from a simple leader model^30^, (p. 62) with the known potential and speed is about three times larger than the value found from the measured current moment with an assumption of the lower end of the leader at 13 km altitude. In addition, for event 1 the leader reaches 42 km altitude and the streamer zone extends to 51 km. If the electric field at the streamer zone boundary is the critical field for negative streamers, the electric field of the leader will probably exceed the breakdown field at 75–85 km altitudes, because it decreases slower than the electrical breakdown threshold field as altitude increases. As a result, high-altitude electrical discharges known as sprites should have occurred, but they did not. We conclude that the electric field in the streamer zone is smaller, and we assume it is the critical field for positive streamers.

With this assumption, the leader tip potential and current of event 1 are estimated to be 10 MV and 35 A, respectively, when it reaches 42 km altitude. The current agrees reasonably well (about 40% larger) with the value derived from the current moment. The two quantities for the event 7 right before the final jump are 28 MV and 180 A, respectively. However, when the leaders just exit from the cloud, their potential and current could be significantly different from those values. According to the binary leader theory of lightning development^30^, (p. 153), a leader acquires an average potential of the thundercloud volume occupied by itself, and as the leader develops its potential may undergo substantial changes. Assuming the lower end of the leader is at 13 km altitude, the leader current for event 1 is ~340 A and the derived potential is 100 MV, when the leader just exits from the thunderstorm. For event 7, they are 270 A and 70 MV, respectively. Surprisingly, the leader of the jet event initially has a larger current and potential than those of the gigantic jet. However, the current moment for the jet leader decreases as the leader propagates upward, while that for the gigantic jet increases. The decreasing current moment while the channel length is increasing means that the current and potential of the leader decrease. With this information, an explanation why the two leaders evolve into the jet or gigantic jet may be formulated as follows. For the jet, the supporting in-cloud positive leader probably stops propagating or extends very slowly; hence, it does not explore an extensive cloud charge region to compensate the decrease in the leader potential due to the extension of the upward negative leader in the low-potential region above the cloud. As a result, the overall potential of the leader decreases rapidly as the upward leader develops. On the other hand, the positive leader for the gigantic jet probably moves through an extensive cloud region and effectively slows down the decrease in the potential due to the extension of the upward leader, allowing it to make the final jump to the ionosphere. Therefore, the dynamic development of both the upward leader and accompanied in-cloud discharges is critical for the formation of starters, jets and gigantic jets. At present, the formation of the upward electrical discharges is best studied by fractal models, but the altitude dependence of the leader channel size is not taken into account by a typical fractal model. Consequently, an escaped upward leader simulated by the fractal model tends to reach high altitudes. An improvement to the fractal model would be introducing the altitude-dependent spatial scale of the leader in the model, so that the potential of the dynamic binary leader system can be more accurately calculated.

## Methods

### Video acquisition and analysis

Supplementary Movie 1 was recorded by a Watec 120N+ camera and Supplementary Movies 2 and 3 were recorded by two Sandia Allsky cameras. All the cameras are coupled to separate triggering systems to record a few-second video when specified trigger criteria are met. The NTSC video from the Watec camera is time stamped by a GPS-synchronized video time inserter. However, a comparison of the timings of the NLDN lightning events and the cloud flashes visible in the videos of the events indicates that the GPS time stamped on the video probably delays by 33 ms (two video fields). This delay has been corrected in Fig. 3. The images shown in Fig. 2 are cropped video fields obtained by deinterlacing the Watec video with FFmpeg software. The raw video data in avi format recorded by the video acquisition system are converted to MP4 format to reduce the data size also by using FFmpeg. The locations and heights of the events were obtained by a combined analysis of the Watec images using information from the star field and NLDN lightning locations. The Allsky system uses a HB-710E Star Light B/W charge-coupled device camera with an auto iris, fisheye lens and the recorded video is stamped with the computer clock time synchronized to the network time.

### Electromagnetic measurements and analysis

Electromagnetic signals radiated by lightning and other natural electrical discharges can be measured either locally or remotely, to extract the information about the source discharges. The horizontal magnetic field waveforms presented in Fig. 3a,c,e were recorded with an LF magnetic field detector, sensitive to the frequency range of 1–300 kHz, at the Florida Tech observation site. At a field site near Duke University, radio emissions in frequency band of 0.1–400 Hz (ULF) are measured with two pairs of magnetic induction coils. The ULF system is ideal to measure slowly varying sources, for instance, the current flowing in a discharge channel between a cloud and either ground (lightning) or the ionosphere (gigantic jets); on the other hand, the LF detector with its higher sensitive frequency range can monitor more rapidly varying sources with a timescale down to a few microseconds, for example, cloud discharges. The current moment (the integral of the current along the entire channel) is extracted from the ULF magnetic field measurements^12^. The cumulative charge moment change can then be obtained by integrating the current moment over the discharge period.

## Author contributions

N.Y.L. drafted the manuscript, supervised the project and analysed the images, storm/lightning data and electromagnetic signals. N.S. operated the Watec camera system, analysed the images and performed the star field analysis. J.R.D. and H.K.R. analysed the images. D.F. operated the two Sandia Allsky cameras and analysed the images. S.A.C collected and analysed the electromagnetic signals. All authors contributed to the discussion of the results and the preparation of the manuscript.

## Additional information

**How to cite this article:** Liu, N. *et al*. Upward electrical discharges observed above Tropical Depression Dorian. *Nat. Commun.* 6:5995 doi: 10.1038/ncomms6995 (2015).

## Supplementary Material

Supplementary Information Supplementary Figures 1-2, Supplementary Note, and Supplementary References

Supplementary Movie 1 Low-light-level video of seven upward electrical discharges above Tropical Depression Dorian. The video is recorded by an automatic video acquisition system located on the roof of a building on the campus of Florida Institute of Technology.

Supplementary Movie 2 The same seven events recorded by a Sandia Allsky camera installed on the same Florida Tech site.

Supplementary Movie 3 The same seven events recorded by another Sandia Allsky camera located about 10 km northwest from the Florida Tech site.
