# Supplementary material for: Upward electrical discharges observed above Tropical Depression Dorian
Source: Nat Commun. 2015 Jan 21;6:5995. doi: 10.1038/ncomms6995 (PMC4354034; doi:10.1038/ncomms6995)
Supplement: Supplementary Information — Supplementary Figures 1-2, Supplementary Note, and Supplementary References [file ncomms6995-s1.pdf]

Supplementary Figures

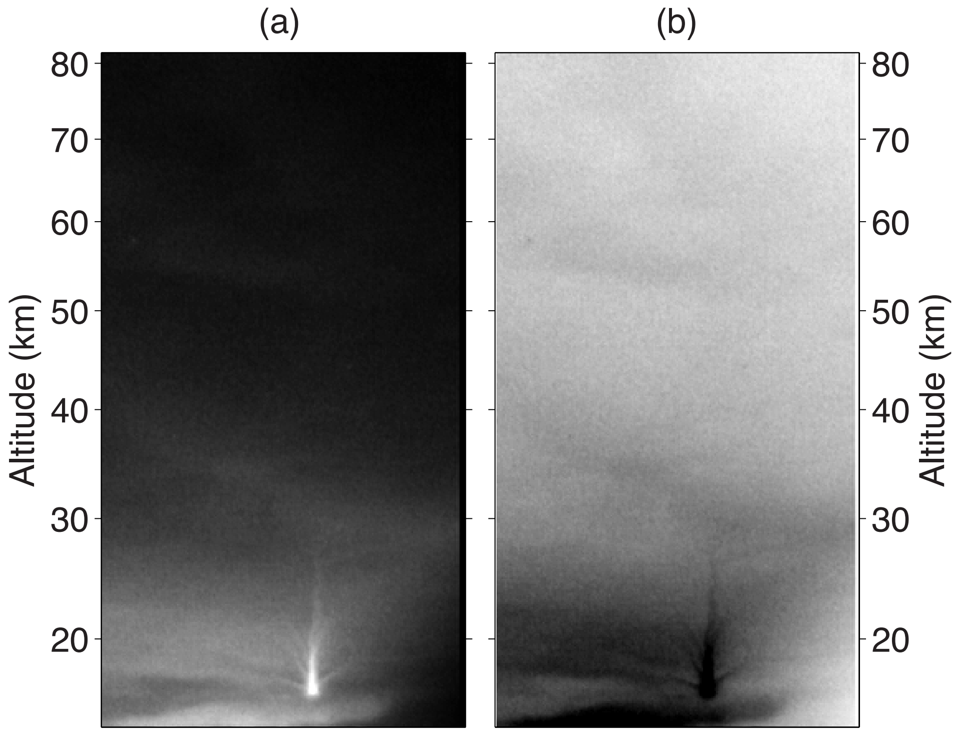

Supplementary Figure 1 | Composite image of the starter event. The composite image is obtained by averaging over 31 consecutive image fields (a time interval of 520 ms) containing the 16 fields of the starter event. (a) a contrast-enhanced composite image of the event and (b) the inverted version of (a). The figure shows that the morphology of the starter is significantly different from that of the positive starter reported previously[1].

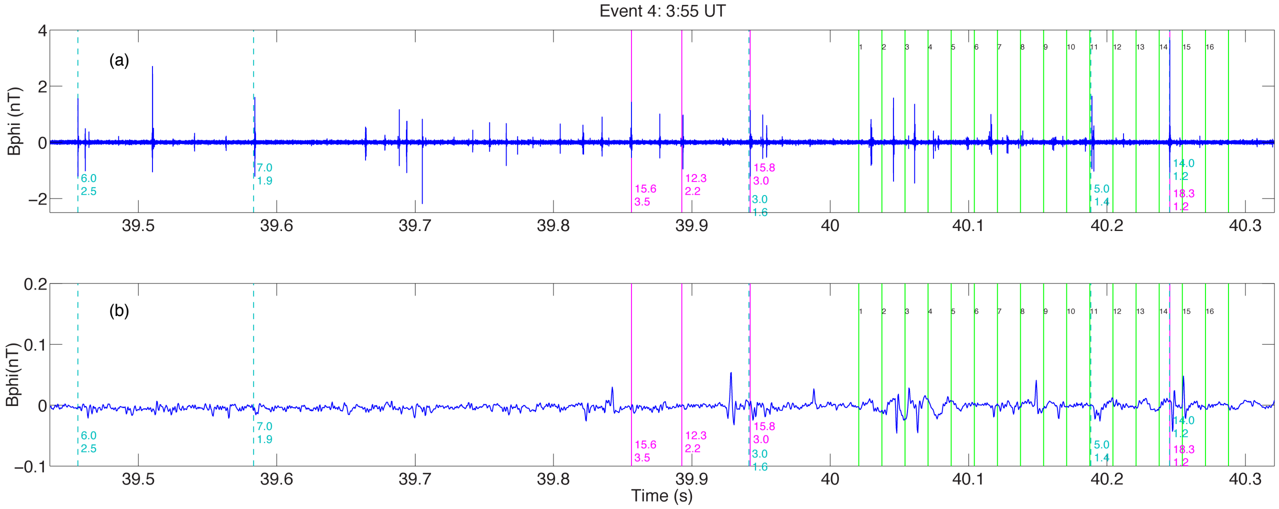

Supplementary Figure 2 | Magnetic field measurements of the starter event. (a) Low frequency and (b) ultralow frequency magnetic field measurements of the cloud discharges associated with the starter. The associated NLDN events and radiation sources detected by the 4DLSS system at Kennedy Space Center are also shown in the figure. The vertical lines in magenta show the timings of the NLDN events, and the upper and lower numbers next to each line are the peak current (kA) and the horizontal distance (km) to the starter. The vertical, dashed lines in turquoise show the timings of the electromagnetic radiation sources detected by the 4DLSS system at Kennedy Space Center. The upper and lower numbers are the source height (km) and the horizontal distance (km) to the starter. There are five NLDN events, and all of them are positive IC flashes. Their polarity is consistent with the polarity of the LF pulses aligned in time. There are four radiation sources detected by the 4DLSS system, and the corresponding LF pulses also show that negative charge is moved upward. For all the NLDN and 4DLSS detections, the associated ULF pulses with negative polarity are discernible. Close inspection of the LF waveform indicates that nearly all the distinct LF pulses during the entire time interval have negative polarity and there are essentially no signatures of downward negative leaders that would be expected to accompany a starter of positive polarity[1–3]. It should be noted that ULF signals can propagate in the earth-ionosphere waveguide for a much longer distance than LF signals. The occasional ULF pulses with positive polarity are produced by some discharges unrelated to the parent storm cell.

Supplementary Note

Fractal modeling has predicted that prior occurrence of intra-cloud (IC) lightning prevents the initiation of electrical breakdown between the upper cloud charge and the screening charge to form starters or jets, while for normal polarity thunderstorms, negative cloud-to-ground (CG) lightning helps produce the electrical breakdown between those two charged layers[2,3]. The positive starter reported previously[1] occurred during a negative NLDN CG flash, confirming the fractal modeling prediction. However, the starter reported here occurred after three NLDN IC flashes, and the LF and ULF waveforms also show there were no CG flashes prior to the starter. Therefore, an interpretation of a positive starter would be inconsistent with fractal modeling results.

Supplementary References

1. Edens, H. E. Photographic and lightning mapping observations of a blue starter over a New Mexico thunderstorm. *Geophys. Res. Lett.* 38, L17804 (2011).

2. Krehbiel, P. R. *et al.* Upward electrical discharges from thunderstorms. *Nat. Geosci.* 1, 233–237 (2008).

3. Riousset, J. A., Pasko, V. P., Krehbiel, P. R., Rison, W. & Stanley, M. A. Modeling of thundercloud screening charges: Implications for blue and gigantic jets. *J. Geophys. Res.* 115, A00E10 (2010).
